# Chromosomal Evolution in Mole Voles *Ellobius* (Cricetidae, Rodentia): Bizarre Sex Chromosomes, Variable Autosomes and Meiosis

**DOI:** 10.3390/genes8110306

**Published:** 2017-11-03

**Authors:** Sergey Matveevsky, Oxana Kolomiets, Alexey Bogdanov, Mikhayil Hakhverdyan, Irina Bakloushinskaya

**Affiliations:** 1 Vavilov Institute of General Genetics, Russian Academy of Sciences, Moscow 119991, Russia; olkolomiets@mail.ru; 2 Koltzov Institute of Developmental Biology, Russian Academy of Sciences, Moscow 119334, Russia; bogdalst@yahoo.com (A.B.); irina.bakl@gmail.com (I.B.); 3 National Veterinary Institute, Uppsala 75189, Sweden; mikhayil.hakhverdyan@sva.se

**Keywords:** *Eif2s3y*, *Sry*, *Ellobius*, karyotype evolution, meiosis, sex determination, tetraploid spermatocytes

## Abstract

This study reports on extensive experimental material covering more than 30 years of studying the genetics of mole voles. Sex chromosomes of *Ellobius* demonstrate an extraordinary case of mammalian sex chromosomes evolution. Five species of mole voles own three types of sex chromosomes; typical for placentals: XY♂/XX♀; and atypical X0♂/X0♀; or XX♂/XX♀. Mechanisms of sex determination in all *Ellobius* species remain enigmatic. It was supposed that the Y chromosome was lost twice and independently in subgenera *Bramus* and *Ellobius*. Previous to the Y being lost, the X chromosome in distinct species obtained some parts of the Y chromosome, with or without *Sry*, and accumulated one or several copies of the *Eif2s3y* gene. Along with enormous variations of sex chromosomes, genes of sex determination pathway and autosomes, and five mole vole species demonstrate ability to establish different meiotic mechanisms, which stabilize their genetic systems and make it possible to overcome the evolutionary deadlocks.

## 1. Introduction

The sex of eutherian mammals is usually determined by the genes maintained in sex chromosomes, XX in females and XY in males. The description of mole voles with odd chromosome numbers, *Ellobius lutescens*, 2n = 17, X0 in males and females [1] was a start of the fascinating saga of sex determination without a Y chromosome in mammals. To date, two rather distinct rodent groups, *Ellobius* and *Tokudaia*, share some intriguing features, such as a loss of Y chromosome in several species and retention of the Y in others. Species *Tokudaia osimensis* and *E. lutescens* have a unique sex chromosome composition XO/XO [2,3,4,5,6,7,8,9,10]. Any genetic differences between males and females have not been confirmed, and even became more intriguing after recent study of the *Ellobius* genome [11] and experiments on *T. osimensis* cells in vitro, which demonstrated high sexual plasticity, reversible differentiation and adaptation from female somatic cells to male germline cells in the male reproductive niche [12]. Three *Ellobius* species, *Ellobius talpinus*, *Ellobius tancrei*, and *Ellobius alaicus*, lost a Y chromosome and obtained isomorphic XX sex chromosomes in males and females, which is a unique case in mammals. Vanishing of a Y chromosome need not necessarily result in the loss of a sex determining factor, which can move to X or autosomes, but usually such cases are known for human pathology, or part of populations in some mammals [4,13]. An absence of the transcription factor SRY (sex-determining region Y) was the first time described in the *Ellobius* species [14]. Later, its absence was also detected for *T. osimensis* [8]. SRY operates by activating the related gene *Sox9*, which suffers from deletions in its enhancer structure (Testis-specific enhancer of Sox9 core, TESCO domain) in all studied species of mole voles *Ellobius*, including species with Y chromosomes [15]. Another deletion was described for the same TESCO domain in spiny mice *Tokudaia* [16,17]. The complicated system of Y and *Sry* loss or presence with possible upregulation of the second gene in sex determination make these rodents a fruitful model for studying the evolution of sex determination in mammals. Unfortunately, *Tokudaia* species are endangered. They inhabit small islands in the Japanese archipelago, which makes any study of their genetics and biology very difficult. *Ellobius* are rather numerous. All species have large ranges and they can be maintained in the laboratory for years.

The taxonomy of *Ellobius* species remained unclear before genetic studies due to their low morphological variability. Presently, they are included in the subfamily Arvicolinae Gray (Microtinae Schrank) [18,19,20,21,22,23]. Mole voles have a cylindrical body, short fur and short tail; large incisors and strong muscles allow them to dig a complex system of borrows, where these animals spend most of their time. Descriptions of chromosome sets were essential for taxonomical revision of the genus, which consists of two subgenera: subgenus *Bramus* with *Ellobius fuscocapillus* Blyth, 1843 (2n = 36, XX–XY) and *Е. lutescens* Thomas, 1897 (2n = 17, X0–X0) and subgenus *Ellobius* with three morphologically cryptic species with sex chromosomes XX in females and males, northern mole vole *E. talpinus* Pall, 1770 s. str. (2n = 54, number of chromosomal arms or number fundamental (NF) = 54), eastern mole vole *E. tancrei* Blasius, 1884, (2n = 54–30, NF = 56), and Alay mole vole *E. alaicus* Vorontsov et al., 1969 (2n = 52–50, NF = 56) [24]. Diploid numbers in *E. tancrei* and *E. alaicus* vary due to Robertsonian translocations (RB translocations) [24,25].

The cross-species chromosome painting appeared to be a precise approach for studying cryptic species, especially in the case of sibling *Ellobius* species. To date, all species were studied, except *E. fuscocapillus*, using painting probes derived from flow-sorted chromosomes of the field vole *Microtus agrestis* [26]. Analysis of chromosome sets of *E. lutescens* (2n = 17) and *E. talpinus* (2n = 54) by comparative chromosome painting revealed a considerable number of rearrangements: at least 31 fusions and seven fissions differentiated the karyotypes of *E. lutescens* and *E. talpinus* from the hypothetical ancestral *Ellobius* karyotype [26]. The 21 *Microtus agrestis* (MAG) autosomal probes revealed 35 conserved segments in the *E. talpinus*, *E. tancrei* and *E. alaicus* genomes. The MAG X chromosome probe exposed signals on both male and female X chromosomes; the MAG *Y* probes did not show any signal [27,28,29,30,31].

Despite intensive study, no data for sex determination factor in *Ellobius* species with X0 or XX chromosomes have been obtained yet. Therefore, the extension of the study of their genomic specificity, karyotype variation, and behavior of sex chromosomes in meiosis is essential. The objectives for this study were to review and enhance data on sex chromosomes and autosome evolution, and obtain more evidence for verifying the hypothesis of independent loss of the Y chromosome in different *Ellobius* lineages.

## 2. Material and Methods

### 2.1. Meiotic Chromosomes

Synaptonemal complex (SC) preparations were made and fixed using the technique described previously [32,33]. Electron microscopy (EM), immunostaining procedure and antibodies used were described in details earlier [34,35,36].

Poly-l-lysine-coated slides were placed in a phosphate buffer saline (PBS) and incubated overnight at 4 °C with the primary antibodies: mouse anti-MLH1, mouse anti-RAD51 rabbit polyclonal anti-SYCP1, rabbit polyclonal anti-SYCP3, mouse anti-phospho-histone H2AX (also known as γH2AFX) (all antibodies from Abcam, Cambridge, UK) and human anticentromere antibody CREST (Fitzgerald Industries International Inc., Concord, MA, USA). Secondary antibody incubations were performed in a humid chamber at 37 °C for 2 h. The slides were examined using an Axio Imager D1 microscope (Carl Zeiss, Jena, Germany).

### 2.2. Sequencing of the Sry, Eif2s3x and Eif2s3y Genes

Primers: The first set of primers for amplifying a 202-bp fragment of the *Sry*-HMG box was taken from Sánchez et al. [37]. Primer sequences were: SRY-HMG-F 5′-GTC-AAG-CGC-CCC-ATG-AAT-GCA-T-3′ and SRY-HMG-R 5′-AGT-TTG-GGT-ATT-TCT-CTC-TGT-G-3′.

The second set of primers was constructed for the shorter fragment of the *Sry*-HMG box, using sequence of the *E. fuscocapillus Sry* gene, which was retrieved from the GenBank (accession number U22443.1): SRY-EfusF 5′-ATG-TTG-TGG-TCT-CGT-GGT-CAG-3′ and SRY-EfusR5′-TAT-CTG-TGC-CTC-CTG-GAA-AAA-TGG-3′.

For sequencing of the *Eif2s3x* (eukaryotic translation initiation factor 2, subunit 3, structural gene X-linked) and *Eif2s3y* (eukaryotic translation initiation factor 2, subunit 3, structural gene Y-linked) genes published primers of Mulugeta et al. [11] were used.

Total DNA was isolated by dual phenol-chloroform deproteinization after the treatment of shredded tissue with proteinase K [38]. To remove RNA from DNA samples, after the first deproteinization stage, they were processed with ribonuclease A and then the phenol-chloroform treatment was repeated. The polymerase chain reaction (PCR) was carried out in a mixture containing 25–50 ng DNA, 2 µL 10× Taq-buffer, 1.6 µL 2.5 mM dNTPs solution, 4 pM of each primer, 1 unit of Taq-polymerase, and deionized water to a final volume of 20 µL. 

The thermal profile started with initial denaturation at 94 °C (3 min) followed by 35 cycles of denaturation at 94 °C (30 s), annealing at 57–63 °C for *Eif2s3x*, or 60–63 °C (1 min) for *Sry*, or 63–67 °C (1 min) for *Eif2s3y*, and extension at 72 °C (30 s); final extension was conducted at 72 °C for 6 min.

Automatic sequencing was carried out using an ABI PRISM BigDye Terminator v. 3.1 kit (Applied Biosystems, Foster City, CA, USA) with an ABI 3500 genetic analyzer at the Core Centrum of Koltzov Institute of Developmental Biology of the Russian Academy of Sciences, Moscow, Russia.

Samples of conserved tissues from the collection of wildlife tissues for fundamental, applied and environmental research of Koltzov Institute of Developmental Biology RAS were used for sequencing (Table S1).

Sequences of gene fragments were deposited in GenBank, accession numbers: *Sry*-HMG box *E. fuscocapillus*, female Seq22576 MF787748; *Eif2s3y* gene fragments: *E. fuscocapillus*, female Seq22576_1 MF796850; *E. lutescens*, male Seq26776 MF796851 (the sequence is equal to male №25157, females №25155); *E. talpinus*, female Seq26915 MF796852; *E. talpinus*, male Seq26910 MF796853; *E. tancrei*, female Seq24889 MF796854; *E. tancrei*, male Seq24913 MF796855; *E. alaicus*, female Seq25605 MF796856; and *E. alaicus*, male Seq25611 MF796857.

Bayesian inference for the data of the *Eif2s3y* sequences of five *Ellobius* species was evaluated in computer program for the statistical phylogenetic analysis MrBayes ver. 3.2 [39]. Final phylogenetic tree images were rendered in FigTree 1.4.3 (http://tree.bio.ed.ac.uk/software/figtree/). The data were executed with 1 million generations, sampling every 1000 generations, with four independent chains and a burn-in of 25%. 

## 3. Results

### 3.1. Autosome Evolution in Ellobius Сryptic Species

**Figure 1:**
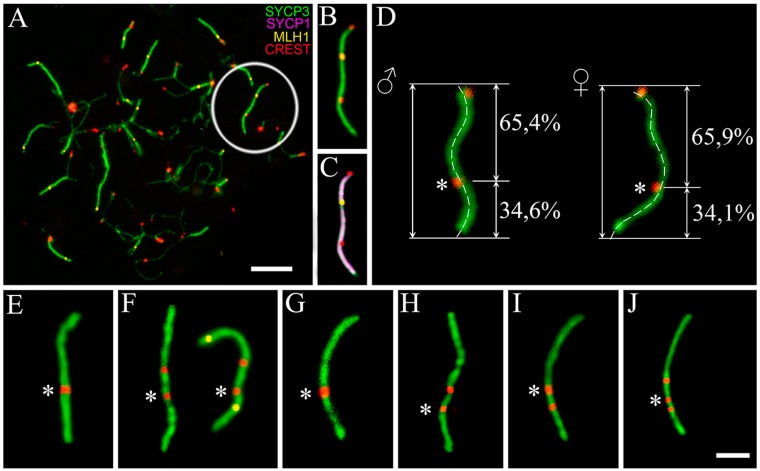
Variations of a centromere position in the submetacentric chromosome in the early meiotic prophase I of mole voles. Axial elements were identified using anti-SYCP3 antibodies (green), central element usinganti-SYCP1 (magenta), recombination nodules usinganti-MLH1 (yellow), and anti-CREST for kinetochores (red). The asterisk shows the location of the centromeric signal of the submetacentric homolog (33–35% of the telomere end). The red signal without asterisk marked erratic centromere positions in the submetacentric. (**A**) spermatocytes from interspecific hybrids F1 of *Ellobius talpinus* (2n = 54) × *Ellobius tancrei* (2n = 34). Bar = 5 µm. A submetacentric is in the circle (inset (**B**–**C**)); (**D**) heteromorphic bivalent in females and males of the interspecific hybrid of mole voles. One centromeric signal originated from the acrocentric homologue of *E. talpinus*, the other from the submetacentric homologue of *E. tancrei*. A submetacentric chromosome from karyotypes of *E. tancrei* 2n = 54, male (**E**); *E. tancrei* 2n = 34, female (**F**); *E. tancrei*, intraspecific hybrid 2n = 50, male (**G**) and female (**H**); *E. tancrei*, intraspecific hybrid, 2n = 49, male (**I**) and female (**J**). Bar (**E**–**J**) = 2 µm.

The species *E. talpinus* and *E. tancrei* have the same diploid number (2n) of chromosomes, 2n = 54, but different fundamental numbers, NF, which are 54 and 56, respectively. *E. tancrei* has one extra pair of submetacentrics in all chromosomal forms (2n = 54–30, NF = 56). Previously, it was assumed that such submetacentrics could arise due to inversion [24]. The most demonstrative test of this assumption could be to study the initial stages of synapsis for these chromosomes in spermatocytes or oocytes of hybrids obtained from the crossbreeding *E. talpinus* and *E. tancrei*. Regarding an inversion, a loop in the meiotic prophase I when chromosome synapsis occurs was observed. Such loop might cover about 2/3 of the bivalent length due to lack of synapsis of acrocentric and submetacentric homologues, which might be easily identified in hybrids, using an electron microscope or fluorescent microscope and immunostaining for SYCP3 (synaptonemal complex protein 3) and CREST (Calcinosis Raynaud’s phenomenon, Esophageal dysmotility, Sclerodactyly, and Telangiectasia) for a centromere region of chromosomes. Earlier, the presence of an inversion loop could not be detected, which suggested that, most likely, an emergence of this submetacentric was due to centromere repositioning [29]. Here, we analyzed >100 nuclei of hybrids from different crossings, and loops or other deviations in the SC structures of this bivalent were never observed (Figure 1А–D). We also observed a single MLH1 (MutL homolog 1) focus (mismatch repair protein that marks the site of recombination) between two centromeric signals in heteromorphic bivalent (Figure 1B,C). Together, these observations support the hypothesis that there is no inversion in the heterobivalent (for more details, see the Discussion section).

The submetacentric had one to three centromeric signals in different chromosomal forms of the *E. tancrei* and hybrids between them (Figure 1E–J). It should be noted that one of the centromeric signals in these pictures was located approximately at the same place as the centromeric signal in the submetacentric homologue of the heteromorphic bivalent in interspecies hybrids (Figure 1D). An additional signal might reflect an unstable state of the neocentromere, which is not fully aggregated yet.

### 3.2. Ellobius Sex Chromosomes: Structure and Meiotic Behaviour

**Figure 2:**
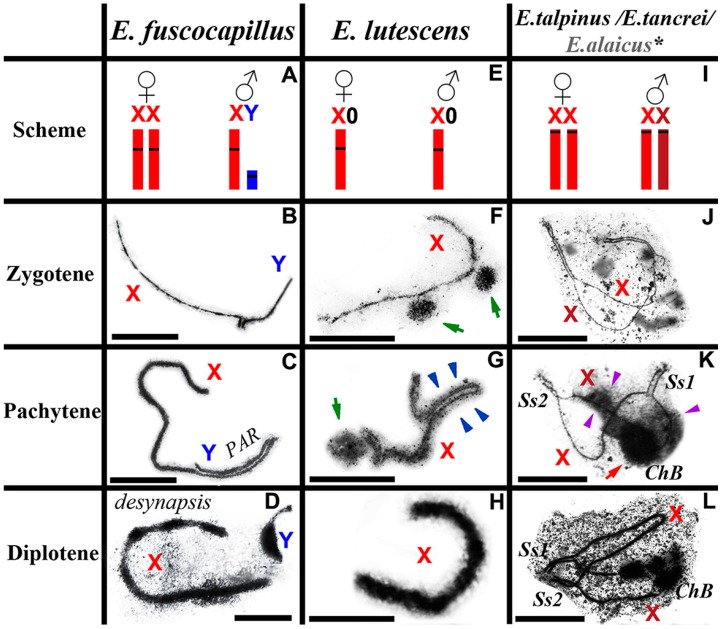
*Ellobius* male sex chromosomes behaviour in meiotic prophase I. Electron microscopy (EM), AgNO_3_-staining (B–D, G–I, L–N). (**А**–**D**) *Ellobius fuscocapillus*. (**A**) sex chromosomes (schematic visualization): ХХ♀/XY♂; (**B**) during the zygotene stage, X and Y synapsed in a short region; (**C**) during the pachytene stage, Y fully synapsed with X. The asynaptic part of the X became thicker; (**D**) during the diplotene stage, X and Y had no synaptic area (desynapsis), chromosomes became thick, irregular and surrounded by an electron-dense cloud; (**E**–**H**) *Ellobius lutescens*. (**E**) sex chromosomes (schematic visualization): Х0♀/X0♂; (**F**) sex Х-univalent was visible as thin axe, one–two round electron-dense bodies often revealed close to it (green arrow); (**G**) during the pachytene stage, an Х-univalent became thicker with multi axes and flexures (‘hairpins’) formed. A large hairpin looked like an SC structure (blue arrowheads). A round body was located nearby (green arrow); (**H**) during the diplotene stage, the X-univalent became thicker; (**I**–**L**) *E. talpinus/E. tancrei/Ellobius alaicus*. Meiosis in *E. alaicus* was still unknown (asterisk). (**I**) sex chromosomes (schematic visualization): ХХ♀/XX♂; (**J**) during the zygotene stage a sex (ХХ) bivalent demonstrated non-standard morphology: two telomeric regions of synapsis and large central asynaptic zone; (**K**) the morphology of ХХ bivalent occurred at the pachytene stage, too. An electron-dense chromatin body (*ChB*) was visualized clearly (red arrow). A less intense electron-dense cloud (purple arrowheads) spread along the X axis from the round body (*ChB*) toward the synaptic sites. Thus, the X axes differ from each other, so Xs are marked with different tones of red; Synaptic sites: *Ss1*, *Ss2* (**L**) during the diplotene stage a sex bivalent rolled up into a tangle and was surrounded by an electron-dense substance. Bar = 5 µm.

The mole voles *Ellobius* maintained a unique diversity of sex chromosomes. *E. fuscocapillus* is the only *Ellobius* species that was characterized by a classical Eutherian system: XY♂/XX♀ (Figure 2A). The X chromosome was a large submetacentric, and Y was a small acrocentric. Electron microscopy (EM) studies of spread spermatocyte nuclei of *E. fuscocapillus* revealed some specific features of the structure and synapsis dynamics of heteromorphic sex chromosomes (X and Y). During the zygotene stage, a short synaptic region formed between the thin and long X and Y chromosomes (Figure 2B). During the middle pachytene stage, Y underwent a complete synapsis with X. The asynaptic part of the axial element of the X became noticeably thicker (Figure 2C). Unlike other mammals, X and Y chromosomes of *E. fuscocapillus* underwent early complete desynapsis during the meiotic prophase I, at the late pachytene-early diplotene, as proposed earlier [7], and this was confirmed with more extensive material (Figure 2D). During the diplotene stage, a compactization of short and thick X and Y chromosomes occurred (Figure 2D).

An unusual sex chromosome system, with a single X chromosome in males and females (X0♂/X0♀) was maintained in *E. lutescens* (Figure 2E). The X-chromosome is large and submetacentric, as in *E. fuscocapillus*, but its Giemsa band (G-band) pattern appeared to be distinct. It was found, by light microscopy [3] and EM [7], that the single X chromosome of *E. lutescens* forms a thickened univalent at the meiotic prophase I. Now, described for the first time at the zygotene-early pachytene stage, one or two electron-dense bodies were positioned close to the thin and long axial of X univalent (Figure 2F). During the pachytene stage, an axial element of the X univalent became thicker, due to a clearer electron microscopy picture, and hairpins could be distinguished in its structure. Moreover, in some long large hairpins, SC-like regions were revealed (Figure 2G). It was clearly seen at the diplotene stage that an axial element of the X chromosome became thicker and obtained a multifibrillar structure (Figure 2H). The male X chromosome formed a typical sex body similar to the XY in mammalian males.

**Figure 3:**
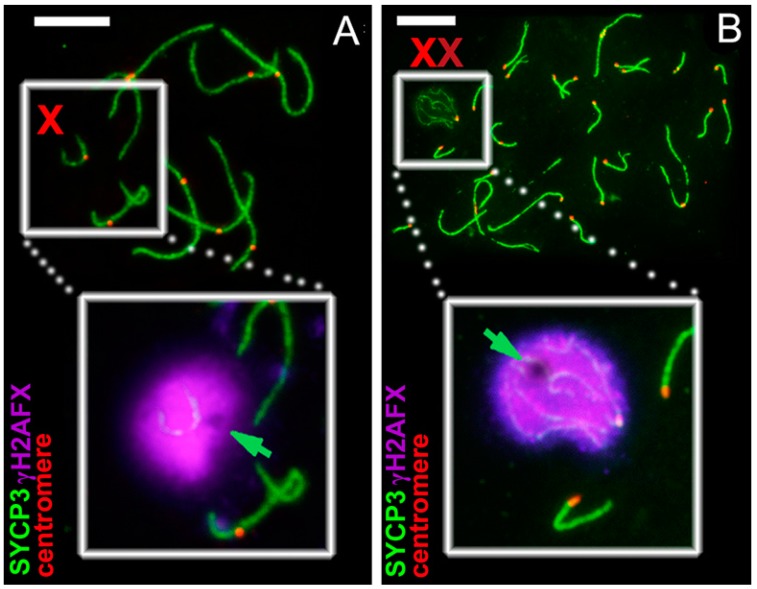
Immunostaining of pachytene spermatocytes from *E. lutescens* (**A**) and *E. tancrei* (**B**). Axial SC elements were identified using anti-SYCP3 antibodies (green), anti-CREST for kinetochores (red) and anti-γH2AFX as marker of chromatin inactivation (magenta). (**A**) the X-univalent is replaced to the periphery of the meiotic nucleus, formed sex body and shrouded by γH2AFX. A γH2AFX-negative round body is noticeable in the X sex body (green arrow); (**B**) during the middle pachytene, XX bivalent was replaced to the periphery of the meiotic nucleus, formed sex body and surrounded by γH2AFX. A chromatin body (*ChB*) was often γH2AFX-negative (green arrow). Bar = 5 µm.

Antibodies to the protein of axial/lateral elements of SC (SYCP3), to the protein of the central element of SC (SYCP1), to the centromeric proteins (CREST), and to the proteins of chromatin inactivation (ATR (ataxia-telangiectasia and RAD3-related), SUMO-1 (small ubiquitin-related modifier 1), histone γH2AFX) were used. During the zygotene stage, γH2AFX signals were detected in unpaired regions. During the pachytene stage, the X chromosome was shifted to the periphery of the nucleus. The ATR- and SUMO-1 signals were immunodetected only within chromatin of the sex univalent (unpublished data). The γH2AFX signals were visualized in chromatin of the X univalent and as specific foci within SC (Figure 3A). Within the chromatin of the X univalent, the electron-dense, DAPI-positive, γH2AFX-negative round body became visible (Figure 3A). The body was of the chromatin nature in *E. lutescens*, as in other mole vole species, as suspected [35].

Sibling species *E. talpinus*, *E. tancrei* and *E. alaicus* maintained a unique system of sex chromosomes, XХ♂/XХ♀ (Figure 2I). Their X chromosomes were large acrocentrics with identical G-band patterns. Starting from the zygotene stage, the X chromosomes synapsed between the short telomeric regions (Synaptic sites; Ss1, Ss2) and remained asynaptic in the central zone (Figure 2J,K) [7,34,35,40,41]. Starting from the late zygotene stage, in the structure of the sex bivalent, a rounded electron-dense chromatin body intensively stained by AgNO_3_, formed. Sometimes, a less intense electron-dense cloud spread along one of the *x*-axes from the round body (ChB) toward the synaptic sites (Figure 2K). The shape and argyrophility of this round structure led to the conclusion about the nucleolar nature of this structure, previously denoted as a nucleolus-like body. Later, by the immunofluorescence studies, it was found that this structure binds intensely to DAPI, resulting in the assumption that it was DNA. Chromatin inactivation was believed to occur due to the density of this structure [35]. However, it became clear that, after immunostaining of the spread nuclei with antibodies to the histone γH2AFX, this structure appeared to be often (but not always) γH2AFX negative (Figure 3). These results encourage continued research on the issue.

Some other features of recombination of the XX chromosomes were revealed. Recombination nodules formed in the telomeric regions of the bivalent within a very short time. During the pachytene stage, the sex (XX) bivalent gradually displaced the periphery of the nucleus and formed a typical sex body (Figure 3B).

### 3.3. Tetraploid Cell in Ellobius talpinus

**Figure 4:**
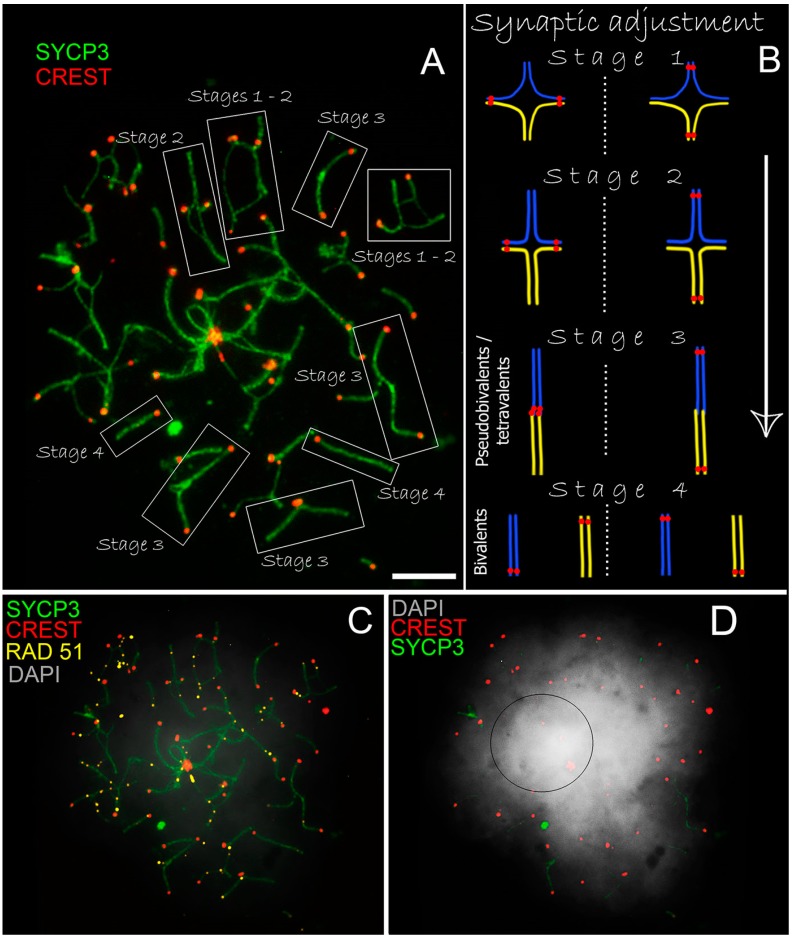
Tetraploid early pachytene spermatocyte from *E. talpinus* with stable karyotype (2n = 54, NF = 54). Axial SC elements were identified using anti-SYCP3 antibodies (green), anti-CREST for kinetochores (red), DNA double-strand break (DSB) loci immunostained with antibodies against the RAD51 protein (yellow). Chromatin was stained with DAPI (gray). Pseudo-bivalents (tetravalents) at the different stages of synapsis were in the nuclei (**A**). Four homologous adjusted sequentially, forming double bivalent/pseudo-bivalent (tetravalent) and then individual bivalents (**B**). RAD51-signals are absent in some fully synaptic bivalents (**C**). The denser chromatin occupied the central part of the nucleus, where it is assumed a sex tetravalent was located (a black circle, (**D**)). Bar = 5 µm.

One of the northern mole vole males had a tetraploid spermatocyte at the early-middle pachytene stage found among more than 400 analyzed cells (Figure 4A–D). More than 50 centromeric signals and pseudo-bivalents (tetravalents) were detected in the nucleus (Figure 4A). Four homologues synapsed with each other and underwent four stages of synaptic adjustment (Figure 4B). The chromosomes started synapsis from the distal ends (stage 1), the synapse then spread over all arms and two opposed homologs synapsed faster (stage 2), and eventually a fully synapsed pseudo-bivalent with a thickened site in the center and two centromeres (stage 3) was observed. Two bivalents were formed from the pseudobivalent (stage 4). The sex bivalent in this nucleus could not be identified, but it might be in the central part, where the densest chromatin was observed (Figure 4C,D). The γH2AFX-immunostaining did not reveal any signals in this nucleus.

### 3.4. Existence of Sry and Eif2s3y Genes in Ellobius

*Ellobius alaicus* was the last species of the genus, for which presence or absence of the *Sry* gene was unknown. The set of published primers for voles was used, and a new set was designed (see Methods) for detecting the *Sry* gene in all *Ellobius* species, males and females. Agreeing with previous analyses [10,11,14], no *Sry*-like sequence was detected in male and female genomes of *E. lutescens*, *E. tancrei*, *E. talpinus*, and, for the first time, for *E. alaicus* (Table S1). Contrasting these results, fragments of the high mobility group (HMG) box of the *Sry* gene were detected in males, and in some females of *E. fuscocapillus* (Table S1, S2). Short fragments of the HMG box in all studied samples of *E. fuscocapillus* (138 bp) were detected, except in one female (21463), and more, longer fragments (203 bp) in another female (22576, GenBank accession number MF787748). More specimens need to be studied to answer the question about the number of copies, which might differ in length and changes.

To track the evolutionary history of the sex chromosomes in *Ellobius*, homologous spermatogonial proliferation factor *Eif2s3x* and *Eif2s3y* genes were used. Fragments of the genes were sequenced and used as markers for phylogenetic reconstructions. The primers of Mulugeta et al. [11] were used and obtained comparable results for *Eif2s3x*, up to identifying 93–94% with *Mus musculus* in all species, except *E. fuscocapillus* (Table S1). The short exonic fragment (162 bp) of *Eif2s3x*, which was sequenced in *E. fuscocapillus*, carried a single transversion (A–T). The BLAST analyses (https://blast.ncbi.nlm.nih.gov) for presumably an exonic part of *Eif2s3y*, revealed up to 88% identity for the studied fragment of *Eif2s3y* of Muennik’s spiny rat *Tokudaia muenninki* [42]. The other sequenced part was highly variable in all studied *Ellobius* species, but was identical in males and females. The assumption was made that this part of a gene was an intron, whose fragment (206 bp) in all *Ellobius* species demonstrated a different level of similarity with non-coding transposable short interspersed nuclear elements (SINEs) B2–B4 according to RepeatMasker Database (http://www.repeatmasker.org). The *Eif2s3y* exonic part differed by at least one transition between *E. fuscocapillus* and *E. lutescens*, while the intronic part was more variable. An attempt to assess the level of variation in the studied fragment of the *Eif2s3y* was made. Except *E. alaicus*, bayesian inference revealed a well-supported branching for all *Ellobius* species, joined into two subgenera (Figure S1).

## 4. Discussion

### 4.1. A Centromere Repositioning and Lability in *Ellobius* Karyotype Evolution

The molecular basis for centromere identity remains enigmatic despite its essential functional importance for recruiting kinetochores and orchestrating chromosome segregation [43]. To date, in eukaryotes, no specific DNA sequences of centromeres were revealed. Probably, a centromere is specified by epigenetic mechanisms [44,45]. One of the unifying characteristics of most eukaryotic centromeres, including neocentromeres, is the presence of histone H3 variant, CENP­A (Centromere Protein-A). Moreover, it was proposed recently that CENP-A carries an epigenetic mark for the centromere specification [46,47].

Evolutionarily new centromeres or neocentromeres may be defined as a centromere located at a novel chromosomal position if compared to the ancestral chromosome. Their occurrence may not be ensured by any structural changes, including inversions [48]. Neocentromeres are devoid of satellite DNA and other centromeric repeats, but contain CENP-A. Some cases of de novo centromeres are associated with diseases, and others are proposed to undergo the centromere repositioning, and have an evolutionary impact. The co-existence of both variants of centromeres (old and new) in the same population could be revealed [49]. Changes in the position of centromeres and an emergence of neocentromeres were first described in humans [50]. Later, a significant correlation was revealed for the arising of neocentromeres and diseases, especially cancer [51,52]. Centromere repositioning is a possible mainstream for karyotype evolution in plants [53,54], and some mammals, for example, equids [55,56] and primates [48,57].

**Figure 5:**
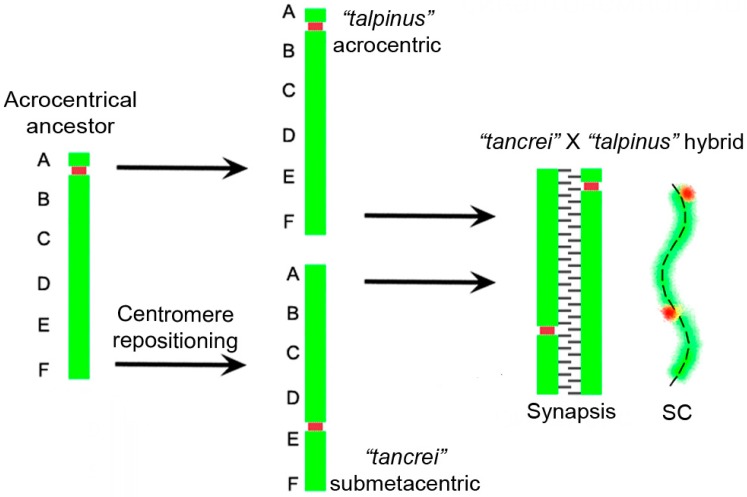
Hypothetical events of chromosome evolution in *Ellobius.* The scheme explains the evolutionary transformation of the ancestral acrocentric into a submetacentric that was inherited by all chromosomal forms of the *E. tancrei*. The synaptic structure at the right is based on the SC structure of the interspecific F1 hybrids of *E. talpinus* (2n = 54, NF = 54) and *E. tancrei* (2n = 54, NF = 56 or 2n = 34, NF = 56; etc.). The red dots indicate centromeres.

In some cases, a linear heteromorphic bivalent can be observed due to linear correction of nonhomologous regions, and subsequent re-synapsis of the axes. The phenomenon is known as synaptic adjustment [58]. Another possible explanation for the case is centromere repositioning or the emergence of an evolutionary new centromere. To confirm or refute the hypotheses, it is necessary to clarify the distribution of recombination sites in the heteromorphic bivalent of interspecies hybrids. No recombination sites between the centromeric signals of the acrocentric and submetacentric homologs of the heteromorphic bivalent will indicate that, despite the inverted arm of the chromosome being paired with the non-inverted one, the synapsis is nonhomologous and recombination is impossible. Earlier, it was shown that the inversion loop did not form, suggesting that the emergence of this submetacentric was most likely due to centromere repositioning [29,59]. New data on immunodetecting of MLH1, which is a crossover marker, allowed for the hypothesis that a recombination nodule formed between the centromeric signals, closer to the centromere of the acrocentric homolog (Figure 1B–D). Thus, absence of synaptic adjustment allowed for the supposition that this submetacentric arose due to de novo formation of the centromere instead of inversion (Figure 5). Inactivation of the old centromere was accompanied by loss or erosion of the near-centomeric heterochromatin [29]. All of these features are typical for the evolutionary new centromeres. Other species, for example, in cucumbers and watermelon, the emergence of new centromeres was accompanied by the attendance of large blocks of heterochromatin and, as in this case, the loss of heterochromatin in the region of the old centromere [54]. Probably, the submetacentric in *E. tancrei* is an evolutionarily young chromosome, and the centromeric heterochromatin is still growing. Consequently, this might be an explanation for the centromere lability, i.e., a phenomenon, when the position of the centromere in the submetacentric is ´not fixed´ strictly. The differences in the form of homologues are apparent at mitotic metaphases. The variability in the position of the centromere was confirmed by observing several centromeric signals in this submetacentric chromosome, when the prophase I of meiosis of different chromosomal forms and intraspecific hybrids of *E. tancrei* was studied (Figure 1E–J). The origin of neocentromere in *E. tancrei* might correlate with its karyotype lability due to Rb translocations. There are no data on any chromosomal changes in *E. talpinus*, despite significant mitochondrial DNA (mtDNA) variability detected [60].

Centromere repositioning in the X chromosome was reported in two species of *Tokudaia* (*T. osimensis* and *T. tokunoshimensis*) with XO/XO sex chromosomes by demonstrating fluorescent in situ hybridization (FISH) mapping of 22 genes [61]. Gene mapping is necessary in the future study of *Ellobius* to establish if the order of the genes on the submetacentric chromosomes was conserved.

### 4.2. Ellobius Chromosomal Variability and Instability: Robertsonian Translocations, Monobrachial Homology and Polyploid Cells

The eastern mole vole *E. tancrei* demonstrates broad chromosomal variations caused by Rb translocations, which changed 2n from 54 to 30. Unlike *Mus domesticus* or *Sorex araneus*, *E. tancrei* obtained numerous Rb translocations in a rather small part of the area in the Pamir-Alay mountains [30,62,63,64]. Applying the G-banding of chromosomes showed a more complex picture of variations, when karyotypes with identical 2n contained distinct metacentrics, homologous inside the Valley of Surkhob River, and non-homologous outside it [65,66].

The next step in studying *Ellobius* was the application of a comparative chromosome painting [27,29,30] that allowed for finding homologies in chromosome structure in species and forms. Unexpectedly, absence of homology in some Rb translocations was revealed, which looked identical in the G-banding pattern [28]. These results resolved a question of why different experimental hybrids demonstrated a distinct decrease in their fertility [65]. The mechanism of that was shown as meiotic aberrations in hybrids with 2n = 49 and 2n = 50 (with four different Rb submetacentrics). These hybrids carried Rb translocations with the monobrachial homology, which resulted in an absence of chromosome synapses in the meiotic prophase I, and an origin of chains, tetravalents and pentavalents [31]. Different numbers and combinations of chromosomes, which were involved in the formation of chains, determined the level of decreasing fertility. An occurrence of associations of sex bivalents with autosomes was revealed in spermatocytes of hybrids with 2n = 49, whereas in females a sex bivalent behaved as autosomes; in other males, and in all types of females, such associations were rare or absent. These deviations may also contribute to the hybrid fertility. This study of experimental hybridization in the mole voles showed that, in the case of monobrachial homology, the semi-sterility of the first-generation hybrids can be overcome, and the number of offspring in next generations can become normal and even larger [28,31,65]. The question of natural hybridization in a case of monobrachial homology is still open because these forms inhabit the opposite banks of the Surkhob River. Many heterozygous animals carrying different numbers of homologous translocations were described. This was a base for the idea of hybridization as a mainstream of *E. tancrei* variability in the Pamir-Alay mountains [66]. Later, it was assumed that, in nature, hybridization occurs between original form (2n = 54) and forms that obtained several Rb translocations, homologous to a low-chromosomal form with 2n = 30 [30]. Other variants need verification by comparative chromosome painting because of low resolution of G-banding. Apparently, there are unstudied regions of the Pamir-Alay where zones of contacts between *E. tancrei* forms with monobrachial homologous chromosome translocations may exist.

Chromosome changes are also known for *E. alaicus* [25], which was originally described as a chromosomal form of *E. tancrei* [24]. *E. tancrei* and *E. alaicus* share one Rb translocation. These species are closely related and may share a genomic predisposition to chromosome changes. 

The model of speciation by centric fusions was proposed by Baker and Bickham [67] for bats. Emergence of monobrachial homology made hybridization difficult and provoked divergence of the forms with such translocations, as was shown in *Mus* [68]. A variant of male and first-generation hybrid sterility and partial female fertility is also known for wallabies of the genus *Petrogale* [69,70]. The contacts between forms with different sets of metacentrics and parental forms became a pathway for the gene flow, but, even in such cases, the decrease of recombination events may result in accumulation of genetic incompatibilities, reproductive isolation, and speciation [71].

It is known that formation of sporadic polyploid cells is possible in humans and animals [72]. Although somatic polyploidy is widely known among animals and humans [73,74], polyploid germ cells are rarely detected. Tetraploid pachytene spermatocytes were detected in mice using EM by Solari and Moses [75] and in humans using immunocytochemical analysis by Codino-Pascual and colleagues [76]. Identified and proved by immunocytochemical study, a tetraploid spermatocyte of the mole vole is the third case of the description of tetraploid cells at prophase I stage among mammals. These unique cases require special attention.

A tetraploid cell arose, probably, during formation of spermatogonia at the mitotic anaphase I stage, if chromosomes could not separate. Such tetraploid spermatogonia might enter meiosis and form a tetraploid spermatocyte I. It was observed that chromosomes tried to form separate bivalents. It is hypothesized that a tetraploid spermatocyte might form diploid spermatozoa, especially since 0.2–0.3% of all spermatocytes are diploid in the normal organisms [76]. Following this hypothesis, the fusion of a diploid spermatozoon with a haploid egg can lead to the triploid organism. Meiosis in polyploid animals has specific features, and challenges their evolution [77,78]. The hypothesis of a polyploid origin of mole voles’ karyotypes was previously proposed [79]. However, it was shown later that karyotype evolution of the mole voles did not undergo polyploidization [27]. The detected tetraploid spermatocyte and broad chromosomal variability might be signs of genome instability in *Ellobius*.

### 4.3. Specificity of Y-Linked Genes *Sry* and *Eif2s3y* in *Ellobius*

An attempt to check the previously published data on *Sry* presence in *Ellobius* by sequencing the most conservative part of the gene, the HMG box [80,81], allowed for the supposition that *Sry* is maintained in several copies as a pseudogene in *E. fuscocapillus* male and female genomes.

Multiple copies of the *Sry* gene is a rare event. Such copies were revealed for *Microtus cabrerae* [82,83], in which at least 24 copies in males and females were detected. Recently, copies of the *Sry* gene were found in mice, for specific lines with sex reversal XY females [84]. The functionality of the *Sry* gene in studied samples of *E. fuscocapillus* is uncertain due to changes in its HMG box, but the presence of such a fragment in *E. fuscocapillus* female and male genomes proved a high evolutionary distance between *E. fuscocapillus* and other *Ellobius* species.

Recently, Yamauchi et al. [85] demonstrated that male reproduction can be achieved by only two Y chromosome genes, *Sry* and *Eif2s3y.* The latter has the homologous X chromosome gene *Eif2s3x*. Mulugeta et al. [11] detected the presence of *Eif2s3y* and *Eif2s3x* in male and female genomes of *E. lutescens* and *E. talpinus*. Notwithstanding, Mulugeta et al. [11] were not able to determine any location for these genes, but they showed that genes were modified significantly if comparing *E. lutescens* and *E. talpinus* sequences with mouse or human ones. Moreover, despite detecting a complete *Eif2s3y* gene with introns for *E. talpinus*, they could not assemble this gene for *E. lutescens.* Partial sequences for the gene for all species were accomplished in this study, and these data were used as a marker for phylogenetical assessment. Bayesian inference exposed deep divergence for two subgenera, and closeness for *E. talpinus*, *E. tancrei*, and *E. alaicus* (Figure S1).

*E. fuscocapillus* has *Eif2s3y* sequence in males and females too, proving the assumption about the relocation of some copies of Y-linked genes to the X chromosome. However, any copy of studied *Eif2s3y* fragments in *E. fuscocapillus* and *E. lutescens* were not revealed in this study, as occurred for the *Sry* gene in females. The variations of the *Eif2s3y* sequences appeared to be another argument for the high divergence of two subgenera (*Bramus, Ellobius*) and distinct pathways of the Y chromosome evolution in mole voles (Figure S1).

### 4.4. *Ellobius* Sex Chromosomes: Evolutionary Trends 

Variability of sex chromosomes might have a key role in the evolution of karyotypes [4], or even promote divergence of the major mammal groups [81]. Evolution of sex chromosomes obtaining differences in ancestral isomorphic autosomes, being distinct or heteromorphic ones, has been studied for many decades [86,87,88,89,90,91,92]. Subsequent multiple chromosomal rearrangements and gradual degeneration result in the formation of the Y chromosome [89,91,92,93]. This process should involve a complete or partial loss of the ability of ancestral autosomes to recombination.

The mole voles *Ellobius* remain unique for mammals’ variability of sex chromosome systems and demonstrate distinct pathways and stages of evolution of sex determination. Major features of their sex chromosomes could develop by selective advantages of species-specific meiotic mechanisms.

*Ellobius fuscocapillus* is the only one of five recent species of the mole voles that maintains typical for placentals ancestral-type sex chromosomes, along with uncommon behavior of X and Y in the meiotic prophase I. Their early complete desynapsis at the late pachytene-early diplotene can lead to incorrect chromosome segregation at the anaphase I stage. It was previously suggested [7] that *E. fuscocapillus* probably inherited these features from the common ancestor of the mole voles. The possible explanation for the unique divergence of sex determination mechanisms within the genus *Ellobius* might be a predisposition to unequal segregation in their common ancestor. Such errors could lead to formation of aberrant gametes with 00, X0 and zygotes X0 and XX, and, finally, the possible loss of the Y chromosome in the common ancestor of *E. talpinus*, *E. tancrei*, *E. alaicus* and *E. lutescens* [7]. Meiosis in *E. lutescens*, which possesses a single sex chromosome, resolved meiotic reduction successfully, but zygotic mortality appeared to be 50% due to unsustainability of zygotes with 2n = 16 (no X chromosomes) and 2n = 18 (two X chromosome) [94]. Nonetheless, the case with two X chromosomes was implemented in subgenus *Ellobius*. The only difference in male and female XX chromosomes of *E. talpinus* and *E. tancrei* was revealed in their meiosis. Isomorphic XX chromosomes in males synapsed and recombined in short telomeric regions in the pachytene I, whereas in females they are fully synapsed. Similar chromosome morphology masks the functional heteromorphism of the male sex chromosomes, which can be seen at meiosis [7,34,35]. The presence of two recombination peaks, one at each synaptic site of the sex chromosomes, suggests that the two ancestral X chromosomes were structurally and functionally isomorphic. Later, they underwent a secondary heteromorphization and became functionally different in the central region. The evolutionary conclusion, since ancestral male mole voles had the XY system, is that the loss of the Y chromosome was the primary event, followed by doubling of the X chromosome.

Mulugeta et al. [11] suggested two independent losses of the Y for two studied species, *E. lutescens* and *E. talpinus*, and genetic predisposition toward development of a new sex-determination system in the common ancestor of mole voles. The lack of data for other species could restrict or even lead to an incorrect interpretation of the obtained results. Here, data are presented on meiotic behavior of sex chromosomes for four species. These results challenged the opinion that the Y chromosome was lost in the common ancestor of all *Ellobius*. Earlier, it was supposed [35,59], and, now, more evidence has been established for an independent loss of Y chromosomes in *E. lutescens* and in the subgenus *Ellobius* after their separation. It is believed that two subgenera (*Bramus* and *Ellobius*) developed different pathways of copying Y-linked genes and elimination of the entire Y chromosome. Along with Y chromosome evolution, Xs demonstrate their own changes in subgenera. The subgenus *Bramus* X chromosomes are submetacentrics, albeit *E. lutescens* keeps a single X in both sexes, while *E. fuscocapillus* maintains the XX–XY system.

Fragments of the *Eif2s3y* as marker for tracking the evolutionary history of sex chromosomes was used. It is possible, that, despite the loss of the Y chromosome as an independent unit, a fragment of the Y chromosome was relocated into the X chromosome. This study detected *Eif2s3y* in males and females with isomorphic XX. These data are considered a confirmation of the assumption that the X was doubled in the subgenus *Ellobius.* Consequently, it cannot be confirmed that gene relocation took place in the common ancestor of all *Ellobius* species. Moreover, the distinctness of the *Eif2s3y* structure within the genus, and the presence of several copies of studied fragments of *Eif2s3y* in the subgenus *Ellobius*, favor independent events of relocation of the gene in distinct species. Absence of *Sry* in males of four species and presence in males and females of *E. fuscocapillus*, along with variations of the *Eif2s3y* structure between species, but not between males and females, and existence of its multiple copies in the subgenus *Ellobius* might indicate independent relocations of Y fragments into X chromosome in two subgenera.

**Figure 6:**
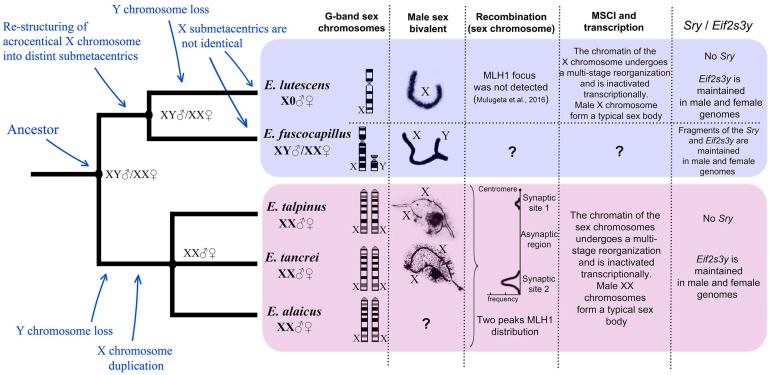
Evolutionary patterns of sex chromosome composition. G-Band: Giemsa band; MSC: meiotic sex chromosome inactivation.

Evolution shaped the morphology of X chromosomes differently in mole voles. For recent subgenus *Bramus* species, *E. fuscocapillus* and *E. lutescens*, Xs have submetacentrics with a distinct number and density of the G-bands [4,7]. The subgenus *Ellobius* has an acrocentric X, which presumably doubled, and three species obtained a pair of isomorphic XX in both sexes (*E. talpinus*, *E. tancrei*, *E. alaicus*) (Figure 6).

Meiotic behaviour differs significantly in mole voles. The manifestation of typical for XY sex bivalent meiotic patterns in the case of XX male sex bivalent in *E. talpinus* and *E. tancrei* might be the first step in the evolution of isomorphic sex chromosomes into heteromorphic ones. Within *E. fuscocapillus*, at the pachytene, XY chromosomes formed a synapsis in the pseudoautosomal region (PAR), which is typical for the mammalian sex bivalent. The *E. lutescens* X univalent thickened during the prophase I, curved and was shaped differently (Figure 2) [7].

Sex heterochromosomes might arise if recombination is halted between a homologous pair of chromosomes. Suppression of recombination produces distinct differences between the X and Y chromosomes. The non-recombining Y chromosome becomes highly heterochromatic and suffers gene loss [95,96]. This common trend can be illustrated by the meiotic behavior of sex chromosomes in males of two species with isomorphic (XX) sex chromosomes, *E. tancrei* and *E. talpinus*. During the meiotic prophase I, a border of the region of suppressed recombination in the sex bivalent was marked sharply from the zygotene to the diplotene stage, while asynaptic zones showed no signs of adjustment. The chromatin of the asynaptiс areas underwent inactivation. Recombination nodules were detected in two short synapsed telomeric parts only. Therefore, a relationship between suppression of recombination and prevention of adjustment between sex chromosomes was revealed for isomorphic sex chromosomes.

## 5. Conclusions

Five mole vole species demonstrate enormous variations of sex chromosomes, genes of sex determination pathway, and autosomes. The only mechanism that enhanced evolution instead of extinction for such living beings is meiosis. The fragile Y hypothesis [97], based mostly on a comparative analysis of Coleoptera, in which loss of the Y chromosome often occurs, proposes an enforced positive selection for gene movement off of the Y chromosome due to translocations or fusions, along with establishing alternate meiotic segregation mechanisms (achiasmatic or asynaptic). Numerous specific traits of sex chromosome behaviour, which were revealed in *Ellobius*, indicated the correlation between the Y loss, gene relocations and emergence of different meiotic mechanisms. Wide karyotypic variability, chromosome instability, lability of centromeres, variety of systems of sex chromosomes and the mysterious process of sex determination without a Y chromosome designate the uniqueness of the genus *Ellobius*, which remains an exclusive object for further evolutionary genetic research.

## Supplementary Materials

Supplementary materials can be found at www.mdpi.com/2073-4425/8/11/306/s1. Figure S1: Bayesian inference for the data of the *Eif2s3y* sequences of five *Ellobius* species was evaluated in MrBayes ver. 3.2.; Table S1: Specificity of the *Sry*, *Eif2s3x* and *Eif2s3y* genes in 5 species of *Ellobius* and GenBank accession numbers; Table S2: Fragments of sequences of *Sry* gene for sex-determining region Y protein and *Eif2s3x,* gene for eukaryotic translation initiation factor 2 subunit 3.
