# Supplementary material for: Chromosomal Evolution in Mole Voles Ellobius (Cricetidae, Rodentia): Bizarre Sex Chromosomes, Variable Autosomes and Meiosis
Source: Genes (Basel). 2017 Nov 3;8(11):306. doi: 10.3390/genes8110306 (PMC5704219; doi:10.3390/genes8110306)
Supplement: Supplementary file 1 [file genes-08-00306-s001.pdf]

Supplemental Information for:

Chromosomal Evolution in Mole Voles *Ellobius* (Cricetidae, Rodentia): Bizarre Sex Chromosomes, Variable Autosomes and Meiosis

Sergey Matveevsky[1]*, Oxana Kolomiets[1], Alexey Bogdanov[2], Mikhayil Hakhverdyan[3], Irina Bakloushinskaya[2]

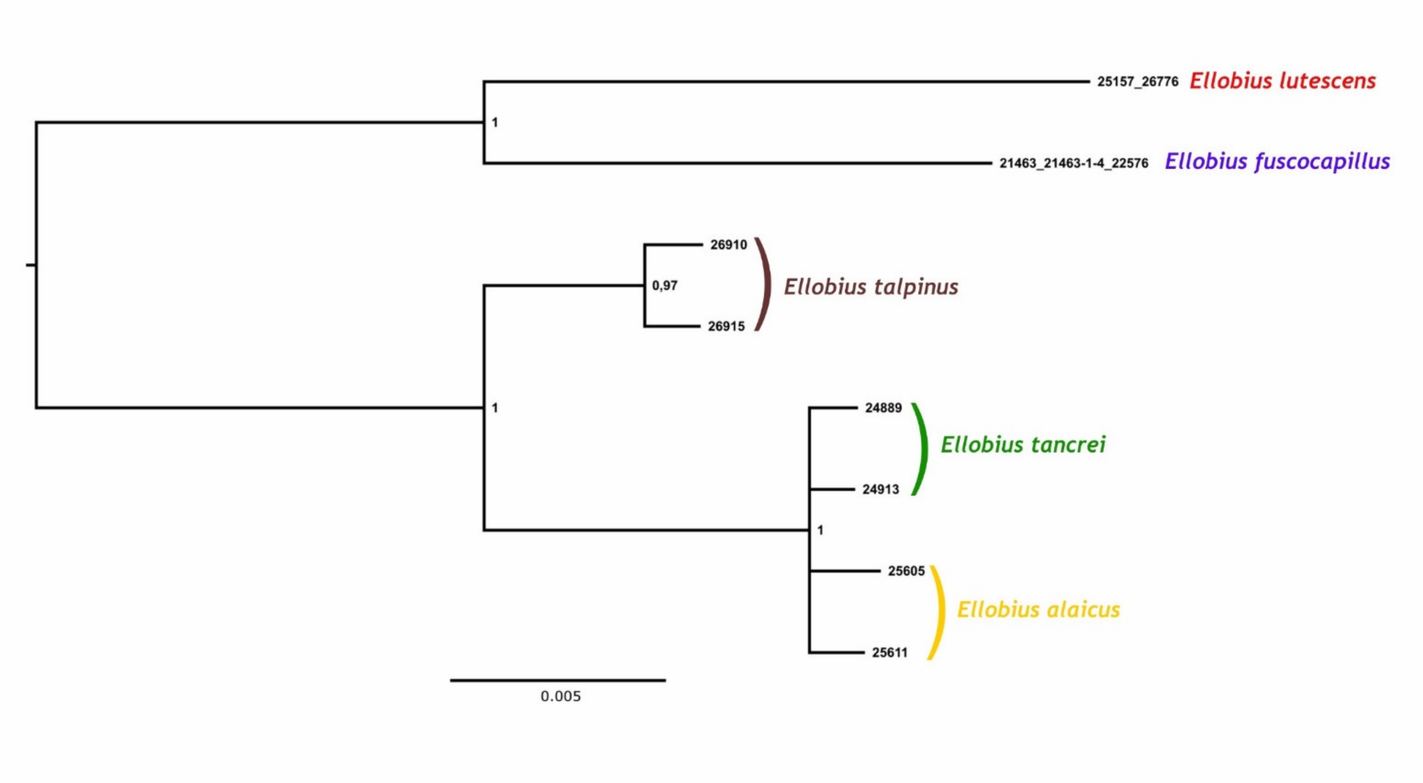

Figure S1. Bayesian inference for the data of the *Eif2s3y* sequences of five *Ellobius* species was evaluated in MrBayes ver. 3.2 (Ronquist et al. 2012). Final phylogenetic trees images were rendered in FigTree 1.4.3. The data were executed with 1 million generations, sampling every 1000 generations, with four independent chains and a burn-in of 25%. Bayesian inference revealed a well-supported tree for all *Ellobius* species, joined into two subgenera.

Reference

Ronquist, F.; Teslenko, M.; Van Der Mark, P.; Ayres, D.L.; Darling, A.; Höhna, S.; Larget, B.; Liu, L.; Suchard, M.A; Huelsenbeck, J.P. MrBayes 3.2: efficient Bayesian phylogenetic inference and model choice across a large model space. *Systematic biology* **2012** *61*(3), 539-542.

Table S1. Specificity of the *Sry*, *Eif2s3x* and *Eif2s3y* genes in 5 species of *Ellobius* and GenBank accession numbers

Species	Voucher number	Sex	Genes			
			Sry full HMG-box predicted length 202 bp	*Sry* fragment of HMG-box, predicted length 144 bp	Fragment of *Eif2s3x*, predicted length 163 bp	Fragment of *Eif2s3y*
E. talpinus	26910	♂	—	—	+ 161 bp	452 bp<br>PCR product was represented by two bands after visualization in gel: major fast, with predicted product size 160 bp and minor slow, with predicted product size 700 bp. The sequence is clearly divided into two parts, major is *Eif2s3y*, and minor is *Eif2s3y* with intron, like in *E. fuscocapillus.* Intron includes fragment of SINEs B2-B4.<br>GenBank accession number MF796853
	26915	♀	—	—	+ 161 bp	521 bp<br>PCR product looked as in 26910<br>GenBank accession number MF796852
E. tancrei	24913	♂	—	—	+ 161 bp	653 bp<br>PCR product looked as in 26910<br>GenBank accession number MF796855
	24889	♀	—	—	+ 161 bp	589 bp<br>PCR product looked as in 26910<br>GenBank accession number MF796854
E. alaicus	25611	♂	—	—	+ 161 bp	537 bp<br>PCR product looked as in 26910<br>GenBank accession number MF796857
	25605	♀	—	—	+ 161 bp	397 bp<br>BLAST to *Tokudaia muenninki* LC066213.1T, EIF2S3Y pseudogenes: 84.2 max score, 74% identity<br>GenBank accession number MF796856

E. lutescens	26776	♂	—	—	+ 161 bp	616 bp<br>The structure of PCR product was similar to that in 26910, but sequences of both parts had many changes<br>GenBank accession number MF796851
	25155	♀	—	—	+ 161 bp	identical to 26776, 25157
	25157	♂	—	—	+ 161 bp	identical to 26776, 25155
E. fuscocapillus	22576	♀	+ 203 bp<br>GenBank accession number MF787748	+<br>138 bp	+ 162 bp	658 bp<br>A specific PCR product about 700 bp divided into two fractions if annealing temperature decreased. An intron was sequenced together with testing fragment of *Eif2s3y*, about 160 bp. Intron included a fragment similar to SINEs B2-B4.<br>GenBank accession number MF796850
	21463	♀	—	—	+162 bp	identical to 22576
	21463-1 embryo	♀ (?)	—	+<br>138 bp	+ 162 bp	identical to 22576
	21463-2 embryo	♂ (?)	—	+	+ 162 bp	identical to 22576
	21463-3 embryo	♀ (?)	—	+<br>138 bp	+ 162 bp	identical to 22576
	21463-4 embryo	♂ (?)	—	+<br>138 bp	+ 162 bp	identical to 22576

Table S2. Fragments of sequences of *Sry* gene for sex-determining region Y protein and *Eif2s3x,* gene for eukaryotic translation initiation factor 2 subunit 3

Genes	Species, voucher numbers	Sequences
Sry-HMG box 138 bp	*E. fuscocapillus* 21463_1-4, 22576	GTTGTGGTCTCGTGGTCAGAGGCGCAAGTTGGCCCTGGAGAACCCCAGC ATGCAAAACACAGAAATCAGCAAACAACTGGGATACCAGTGGAAACGCC TTACAGAAGCCGAAAAAAGGCCATTTTTCCAGGAGGCACA
Eif2s3x 162 bp	*E. fuscocapillus* 21463, 21463_1-4, 22576	AGATCGACCCCACTTTGTGCAGAGCTGACAGGATGGTGGGTCAAGTGCT TGGTGCAGTTGGAGCTTTACCTGAAATATTCACGGAACTGGAAATTTCC TATTTCCTGCTGAGACGTCTCTTGGGTGTACGAACTGAAGGAGACAAGA AAGCAGCAAAGGTCC
Eif2s3x 161 bp	*E. lutescens* 26776, 25155, 25157<br>*E. talpinus* 26910, 26915<br>*E. tancrei* 24913, 24889<br>*E. alaicus* 25611, 25605	GATCGACCCCACTTTGTGCAGAGCTGACAGGATGGTGGGACAAGTGCTT GGTGCAGTTGGAGCTTTACCTGAAATATTCACGGAACTGGAAATTTCCT ATTTCCTGCTGAGACGTCTCTTGGGTGTACGAACTGAAGGAGACAAGAA AGCAGCAAAGGTCC
